# Divergent clades or cryptic species? Mito-nuclear discordance in a *Daphnia* species complex

**DOI:** 10.1186/s12862-017-1070-4

**Published:** 2017-11-22

**Authors:** Anne Thielsch, Alexis Knell, Ali Mohammadyari, Adam Petrusek, Klaus Schwenk

**Affiliations:** 1 Institute for Environmental Sciences, Molecular Ecology, University of Koblenz-Landau, Landau in der Pfalz, Germany; 2 Faculty of Science, Department of Biology, Ferdowsi University of Mashhad, Mashhad, Iran; 3 Faculty of Science, Department of Ecology, Charles University, Prague, Czechia

**Keywords:** Interspecific hybridization, Adaptive introgression, Ancestral polymorphism, Incomplete lineage sorting, Cladocera, *Daphnia longispina* complex

## Abstract

**Background:**

Genetically divergent cryptic species are frequently detected by molecular methods. These discoveries are often a byproduct of molecular barcoding studies in which fragments of a selected marker are used for species identification. Highly divergent mitochondrial lineages and putative cryptic species are even detected in intensively studied animal taxa, such as the crustacean genus *Daphnia*. Recently, eleven such lineages, exhibiting genetic distances comparable to levels observed among well-defined species, were recorded in the *D. longispina* species complex, a group that contains several key taxa of freshwater ecosystems. We tested if three of those lineages represent indeed distinct species, by analyzing patterns of variation of ten nuclear microsatellite markers in six populations.

**Results:**

We observed a discordant pattern between mitochondrial and nuclear DNA, as all individuals carrying one of the divergent mitochondrial lineages grouped at the nuclear level with widespread, well-recognized species coexisting at the same localities (*Daphnia galeata*, *D. longispina*, and *D. cucullata*).

**Conclusions:**

A likely explanation for this pattern is the introgression of the mitochondrial genome of undescribed taxa into the common species, either in the distant past or after long-distance dispersal. The occurrence of highly divergent but rare mtDNA lineages in the gene pool of widespread species would suggest that hybridization and introgression in the *D. longispina* species complex is frequent even across large phylogenetic distances, and that discoveries of such distinct clades must be interpreted with caution. However, maintenance of ancient polymorphisms through selection is another plausible alternative that may cause the observed discordance and cannot be entirely excluded.

**Electronic supplementary material:**

The online version of this article (10.1186/s12862-017-1070-4) contains supplementary material, which is available to authorized users.

## Background

Since the plea for DNA taxonomy [1] and the introduction of the DNA barcoding concept [2] the use of mitochondrial DNA (mtDNA) as genetic marker in animals has become an inherent part not only of taxonomic research but is also widely used in systematics, phylogeography, population genetics, ecology and evolutionary biology. One initial goal of integrating genetic markers into traditional taxonomy was to uncover hidden diversity [2–4]. Especially genetically divergent cryptic species, i.e., those pooled under a single name due to their morphological similarity [5, 6], could be easily detected by the use of molecular markers. Since the introduction of the polymerase chain reaction (PCR) in the 1980s, the number of reports of cryptic species increased exponentially [5].

Interestingly, divergent mitochondrial lineages are still detected even in well-studied groups. This is also true for the freshwater crustacean genus *Daphnia* (Cladocera: Anomopoda), in which previously undescribed lineages were recovered in most biogeographical regions [7]. Within the *Daphnia longispina* species complex, a dominant taxonomic group of *Daphnia* in lake plankton of northern temperate zone, an extensive amount of mitochondrial lineages was reported recently [8–11], with eleven divergent clades without applicable names. The levels of genetic differentiation of seven lineages reported from Europe [8, 11] are as high or even higher than the genetic differentiation among well-recognized species of that complex. Specifically, the sequence divergence from their closest sister clade, expressed as Kimura 2-parameter distance calculated for the mitochondrial gene for the 12S ribosomal ribonucleic acid (12S rRNA), ranged from 10.8 to 17.6% [8]. It was thus suggested that these lineages may represent distinct biological species [8].

However, not every divergent mitochondrial DNA lineage should be considered as species [e.g., [12–14]. Several studies show that the divergence detected at the mitochondrial level is not always supported by nuclear DNA data [e.g., 15, 16]. The reasons for such a discrepancy in mitochondrial and nuclear DNA patterns are diverse [17]. On the one hand, nuclear mitochondrial pseudogenes (numts) or pseudogenes from the mitochondrial genome that originate from duplication events might be amplified rather than the functional target gene [18]. On the other hand, cyto-nuclear discordance may be caused by various biological processes, like hybridization and subsequent introgression of the mitochondrial genome [15, 16, 19], incomplete lineage sorting of ancestral polymorphisms [19–21], or selection acting on mitochondrial genes, either direct due to, e.g., environmental factors [22, 23], or through indirect selection induced by maternally inherited symbionts [reviewed by 24]. Thus, firm conclusions about the delimitations of species based on genetic data should be drawn from a combination of different approaches, e.g., combining mtDNA and nuclear DNA, and assessing evidence on the level of gene flow between putative species.

**Fig. 1:**
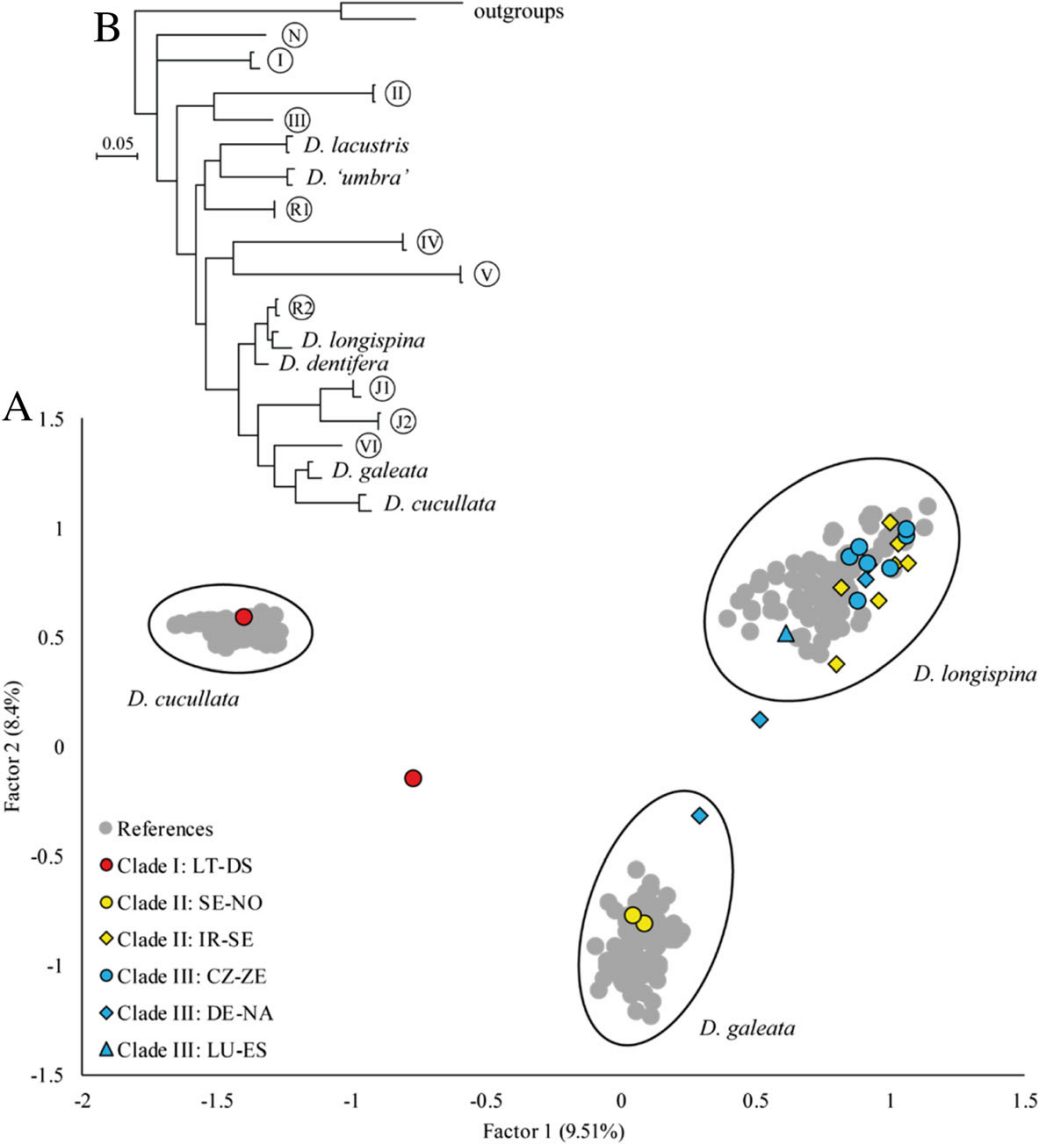
Comparison of nuclear and mitochondrial DNA patterns in the *Daphnia longispina* species complex. Factorial correspondence analysis (**a**) demonstrating the position of the 49 individuals belonging to mtDNA clades I, II, or III in relation to the reference dataset (grey circles) consisting of 312 individuals belonging to *D. galeata*, *D. longispina*, and *D. cucullata*. Genetic relationships are depicted using the first two factors of an FCA based on multilocus genotypes from ten microsatellite loci (see Additional file 1 Fig. S1 for illustrations of factor 1 and 3 as well as 2 and 3). All individuals that were characterized as pure taxa according to their assignment probabilities calculated in Structure 2.3.4 [34] are encased by an oval. Patterns of variation of the 12S rDNA [derived and modified from 8] is shown in the inset Maximum Likelihood tree (**b**). The six divergent mitochondrial lineages firstly described by [8] are labelled I-VI and the remaining five divergent lineages are labelled according to their origin (N: Norway; J1, J2: Japan; R1, R2: Russia)

For most of the unnamed European lineages of the *D. longispina* complex, the available information is scarce, mostly limited to sequences of one or more mitochondrial genes [8, 11]. For one of the lineages from Norway (Fig. 1: lineage N), restriction fragment length polymorphism (RFLP) of the nuclear ribosomal internal transcribed spacer (ITS) suggested its distinctness from other taxa [25]. In contrast, allozyme- and ITS-RFLP-based analyses of *Daphnia* population samples from a Czech reservoir where one of these divergent lineages was later found (Fig. 1: lineage III), did not provide any evidence for the presence of a previously undiscovered species [11, 26]. However, as the samples from that reservoir were analyzed for a different purpose, it is not known whether any individual of this divergent mitochondrial lineage was actually processed in the previous studies [11, 26].

The main aim of our study was to analyze at least some of the distinct mitochondrial lineages of the *Daphnia longispina* species complex reported recently from Europe [8], to gather additional evidence in favor or against the hypothesis that they represent cryptic species. Therefore, we studied individuals belonging to three distinct mitochondrial lineages from six geographically distant localities using nuclear microsatellite markers [27] in addition to the mitochondrial gene for the 12S rRNA.

## Methods

We analyzed 49 individuals of the *Daphnia longispina* complex from six different lakes (Table 1) that belonged to one of the previously undescribed lineages [reported in 8]. While some DNA samples used in the previous study [8] were reanalyzed at the nuclear level (lineage I: lake Druksiai, Lithuania; III: Želivka reservoir, Czechia), further samples were collected from Želivka reservoir and from lake Norrviken, Sweden (lineage II) for analysis of mitochondrial and nuclear DNA. In addition, individuals belonging to some of the previously undescribed lineages were discovered in further localities (Seragah, Iran; Neuhofener Altrhein, Germany; and Esch-sur-Sûre, Luxembourg), and these were included in our analysis as well.

**Table 1:** Data on populations from which analyzed individuals belonging to mtDNA clades I, II and III were obtained.

mtDNA Clade	Population	Country	Location	Latitude (N)	Longitude (E)	Month-Year (N_all_/N_div_)	Coexisting taxa
I	LT-DS	Lithuania	Lake Druksiai	55.63	26.59	09–2006 (49/3)	*D. longispina* *D. galeata* *D. cucullata*
II	IR-SE	Iran	Lake Seragah	37.83	48.88	04–2013 (34/8)	*D. longispina*
	SE-NO	Sweden	Lake Norrviken	59.46	17.93	08–2012 (26/3)	*D. galeata* *D. cucullata*
III	CZ-ZE	Czechia	Želivka reservoir	49.72	15.10	07–2010 (48/7)	*D. longispina* *D. galeata* *D. cucullata*
	DE-NA	Germany	Neuhofener Altrhein (oxbow lake)	49.43	8.46	04–2013 (45/1) 05–2013 (48/2) 06–2013 (146/12) 07–2013 (46/3) 08–2013 (54/9)	*D. longispina* *D. galeata* *D. cucullata*
	LU-ES	Luxembourg	Esch-sur-Sûre reservoir	49.90	5.88	09–2006 (12/1)	*D. longispina* *D. cucullata*

Identification codes of populations are given together with country of origin, location name and associated geographical coordinates. For each location, sampling month as well as corresponding number of all individuals analyzed with 12S rDNA (N_all_) and number of individuals belonging to one of the divergent mtDNA lineages (N_div_) that were subsequently analyzed at microsatellite loci, and the names of coexisting taxa of the *D. longispina* complex, are listed

As no distinguishing morphological characters were reported for the respective mitochondrial lineages, we screened for these lineages by sequencing mitochondrial 12S rDNA in randomly selected individuals from the respective populations (in total, 508 individuals were studied at the mitochondrial level; for more information on sample sizes per population see Table 1). Total DNA was extracted either by using HotSHOT [28] or H3 extraction method [according to 29].

We aimed for the amplification of an approximately 600 bp fragment of the mitochondrial gene for 12S rRNA [following 11]. If DNA samples had low quality and the amplification of the target marker failed, shorter fragments that still allowed discrimination within the *Daphnia longispina* complex were amplified, either according to a previously published protocol [30; fragment length approximately 150 bp] or using newly designed primer pairs (segment 1 with an approximate length of 190 bp: forward 5′ CAGGGTATCTAATCCTGG 3′, reverse 5′ GCGACGGCTGGCACGATTT 3′; segment 2 with an approximate length of 230 bp: forward 5′ CTGCACCTTGACCTGAAGT 3′, reverse 5′ CAGGGTATCTAATCCTGG 3′). All amplification products were sequenced using forward and reverse primer on a capillary sequencer (either 3130xl or 3730 Genetic Analyzer; Applied Biosystems). The sequences were manually checked using Geneious 4 (Biomatters Ltd) with a particular focus on possible presence of double peaks or other ambiguities, and aligned using MUSCLE algorithm [31] implemented in MEGA 6 [32].

Individuals which belonged to one of the previously undescribed mtDNA lineages (clade I = 3 individuals, clade II = 11 individuals, and clade III = 35 individuals) were subsequently analyzed at the nuclear level by characterizing the variation at ten microsatellite loci (DaB17/17, Dgm105, Dgm109, Dgm112, Dp196NB, Dp281NB, Dp519, SwiD6, SwiD14, SwiD18) that were originally developed for *D. galeata*, *D. longispina*, *D. cucullata* and other taxa of the *D. longispina* complex [27, 33]. Each individual was assigned to a multilocus genotype (MLG), based on the allelic variation at all ten loci. We compared the allelic variation with a reference dataset consisting of 312 individuals of *D. galeata*, *D. longispina*, and *D. cucullata* from 21 European locations [see Additional file 1: Table S1 for information on reference populations; partly published in 33].

To test if individuals with previously undescribed mtDNA lineages were also divergent at the nuclear level, we used a model-based assignment test implemented in Structure 2.3.4 [34] to identify the number of genetic clusters in the total dataset (i.e., 312 reference individuals and 49 individuals belonging to the three divergent mitochondrial lineages). As we were only interested in the interspecific patterns, we limited the number of expected groups to a range of *K* = 1–10 (the upper bound already exceeding the number of expected taxa within the dataset).

For each value of *K* we conducted ten independent replicates using 100,000 burn-in iterations and 900,000 Markov Chain Monte Carlo (MCMC) steps and we used the admixture modus (ancestry model) as well as independent allele frequencies (allele frequency model). For the assessment of the most likely value of *K*, we employed the Δ*K* method [35] implemented in Structure Harvester [36] and evaluated Ln P(D), convergence between replicates as well as individual assignment probabilities. Individuals with a probability to belong to a certain cluster exceeding 90% were regarded as pure-taxon individuals while the others were regarded as hybrids or individuals showing some level of backcrossing or introgression [according to 37]. In addition to the model-based assignment test, the pattern of similarity among individuals from the whole dataset, based on their MLGs, was characterized using an factorial correspondence analysis (FCA) implemented in Genetix 4.05 [38].

Furthermore, we conducted a hierarchical Structure analysis using all individuals assigned to *D. longispina*, including 103 reference individuals, eight individuals belonging to clade II and nine individuals belonging to clade III. The number of *K* was set to 1–15 with ten replicates for each *K* using 50,000 burn-in iterations and 450,000 MCMC steps for each replicate. As in the preceding Structure analysis description, we used the admixture modus as well as independent allele frequencies among populations and employed Δ*K*, Ln P(D) and convergence between replicates to assess the most likely number of *K* in the *D. longispina* data set. With this additional analysis, we wanted to evaluate if within one taxon a substructure is apparent that would support the distinct status of the divergent lineages.

## Results

We sequenced 508 individuals from the six studied localities and detected 49 individuals belonging to one of the previously undescribed mtDNA clades (Table 1). Three were assigned to clade I (Druksiai, Lithuania), eleven to clade II (three from Norrviken, Sweden; and eight from Seragah, Iran), and 35 to clade III (seven from Želivka, Czechia; 27 from Neuhofener Altrhein, Germany; and one from Esch-sur-Sûre, Luxembourg). Clades I and III were only represented by a single already reported haplotype [8]. Clade II, however, was represented by two haplotypes, one found in the Swedish population [8] and the other one in the Iranian population (GenBank accession number: KY652589). In all locations, other coexisting *Daphnia* taxa were detected when sequencing the mitochondrial 12S rDNA (Table 1); in three sites (Druksiai, Želivka, and Neuhofener Altrhein), three different hybridizing species coexisted (*D. longispina*, *D. galeata*, and *D. cucullata*).

**Fig. 2:**
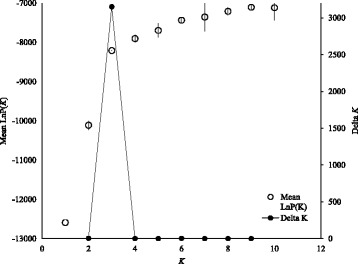
Detection of the uppermost hierarchical level of genetic structure in the complete microsatellite dataset. Ln P(D) and convergence between replicates (open circles with error bars) as well as Delta *K* (filled circles) were used for the detection of the most likely number of *K*. The graph is modified from the output derived from Structure Harvester [36]. According to these results, *K* = 3 is adequate to describe the structure in the dataset

**Fig. 3:**
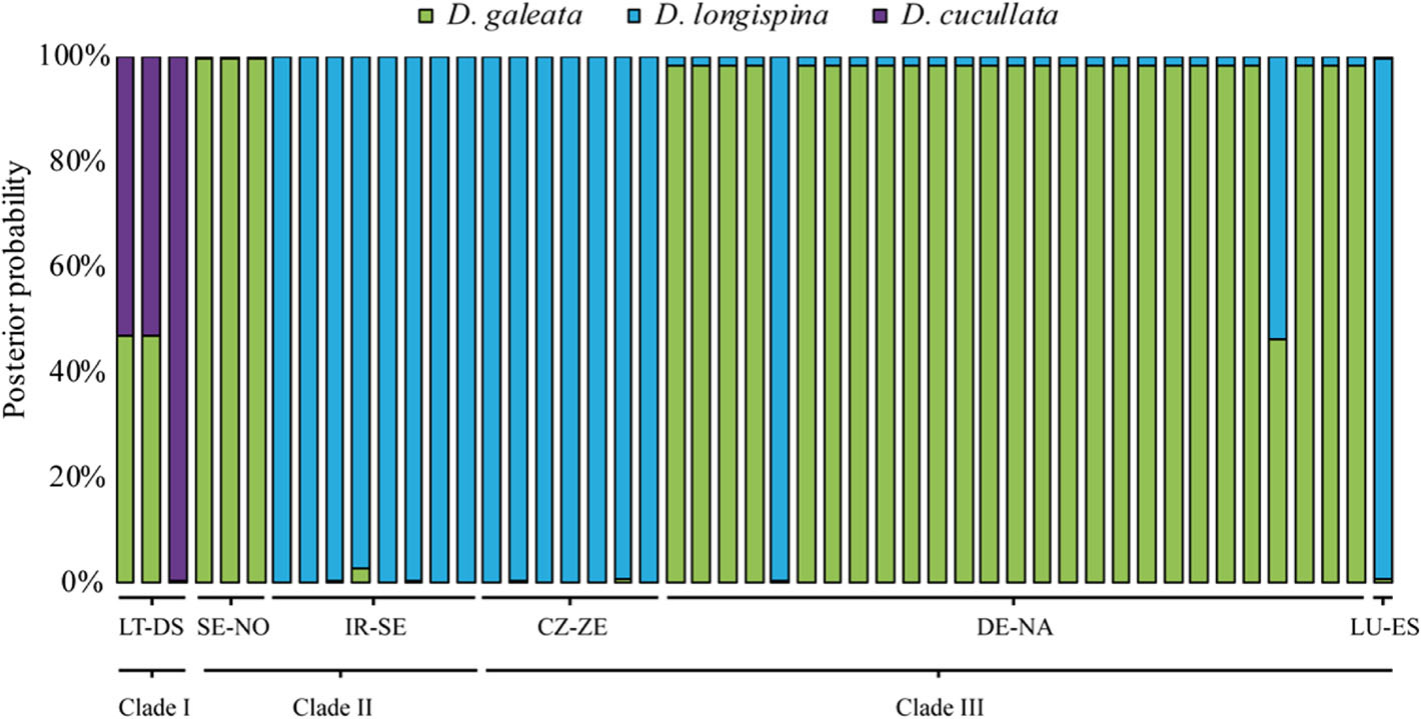
Results of the admixture analysis of all 49 individuals belonging to mtDNA clades I, II, and III. Analysis was performed using Structure 2.3.4 [34] with *K* = 3 where each cluster associates to one of the well-recognized species *D. galeata* (green), *D. longispina* (blue), and *D. cucullata* (purple); reference data are not shown. The population identification code as well as the corresponding mtDNA clade are given on the x axis and the posterior probabilities on the y axis. For information on population codes see Table 1

All microsatellite loci amplified well in all three divergent mtDNA clades. The clustering approach used by Structure 2.3.4 [34] suggested that *K* = 3 was appropriate for our dataset based on Evanno’s Δ*K* [35] as well as the evaluation of Ln P(D) and the convergence between replicates (Fig. 2). The three clusters were associated with the three widespread species of the complex, *D. galeata*, *D. longispina*, and *D. cucullata*, and the 49 individuals belonging to mtDNA clade I, II, or III assorted to either one of the above-mentioned species or exhibited an admixed genotype indicative of individuals with hybrid status (Fig. 3). The factorial correspondence analysis (Fig. 1, Additional file 1: Fig. S1) supported this pattern as well. All 49 individuals grouped within the clusters representing *D. galeata*, *D. longispina*, and *D. cucullata* or their hybrids.

Analysis of the structure within *D. longispina* (including 103 reference individuals and individuals of clade II and III) resulted in a most likely number of seven or eight clusters, respectively (Additional file 1: Figure S2). These clusters corresponded mainly to geographically distinct populations, and the resulting pattern did not support a separation of *D. longispina*, clade II and clade III (Additional file 1: Figure S3).

## Discussion

The distinctness of the 49 focal individuals, each belonging to one of the three studied mtDNA clades according to 12S rDNA, was not confirmed at the nuclear level. On the contrary, all these individuals were assorted to one of the coexisting species of the complex (Fig. 1): *D. galeata* (clade II), *D. longispina* (clade II and III), and *D. cucullata* (clade I). This finding explains the lack of earlier nuclear evidence (based on allozyme and ITS-RFLP studies) of an unknown taxon in the reservoir Želivka [see 8]. An additional study analyzing in detail *Daphnia* population structure from the same locality by ten microsatellite markers also failed to find any evidence of an additional distinct taxon [39]. Our data also show that the divergent mtDNA lineages are not a short-term phenomenon but rather persist over longer time periods: Lineage III was detected at different times over one growing season in Neuhofener Altrhein (Table 1) and lineage II was still detected in lake Norrviken after almost two decades.

The reason for these discordant patterns of mitochondrial and nuclear DNA could be, in principle, a methodological artifact in case we failed to amplify the functional mtDNA gene under study and sequenced pseudogenes instead. Especially, the so-called numts (nuclear copies of mitochondrial DNA) are reported frequently [18]. These numts are, in general, observed for protein-coding genes [but see 40 for an example of ribosomal DNA] and some of them still look functional (i.e., exhibit no signs of stop codons or frameshifts within the coding region). In addition, cases are known where those numts are diverged enough that they were inappropriately considered as evidence for the presence of different species [see 18 for more information]. Besides numts, mitochondrial pseudogenes due to gene duplication have also been observed [41] but they are assumed to be very rare.

However, we consider the pseudogene scenarios an unlikely explanation for the observed patterns. First, the original study reporting the divergent lineages [8] already evaluated whether the secondary structures of obtained sequences correspond to that expected from functional ribosomal RNAs. Second, for some individuals from the reservoir Želivka [8] and the lake Seragah (unpublished data), additional partial sequences of mitochondrial genes for the cytochrome c oxidase subunit 1 (COI) and for the reduced nicotine adenine dinucleotide dehydrogenase subunit 2 (ND2) were obtained, confirming the distinctness of the mitochondrial genomes of those lineages.

There are, however, further explanations for the discordant patterns of mitochondrial and nuclear DNA that should be considered when evaluating the hypothesis that divergent mitochondrial lineages represent cryptic species within the *D. longispina* complex.

For example, cases of mito-nuclear discordances are observed after hybridization and subsequent introgression of the mitochondrial genome [15, 16, reviewed by 17]. The authors of a recent meta-analysis on freshwater and marine fish emphasized that discordance is expected to be higher in freshwater fish due to greater historical hybridization and introgression within this group [42]. Individuals of the genus *Daphnia* and especially of the *D. longispina* complex are known to hybridize regularly [43]. In the *D. longispina* complex, *D. galeata* is reported as the most “aggressively” hybridizing taxon [44] and its interspecific hybrids with at least five other taxa (*D. mendotae*, *D. lacustris*, *D. dentifera*, *D. longispina*, and *D. cucullata*) have been detected [e.g., 9, 33, 45, 46]. Most hybrids of the *D. longispina* complex are categorized as F_1_ hybrids, but also later-generation hybrids and backcrosses have been frequently detected [e.g., 33, [47–50], which confirms that gene flow between the parental species, although reduced [51], is not entirely restricted. This is also supported by studies that detected mitochondrial introgression within the *D. longispina* complex [50, 52–54] as well as mitochondrial capture in other *Daphnia* species [55]. Thus, the discordant patterns found in our study may indeed be explained by introgressive hybridization. However, the source taxa of the mitochondrial genomes are not known, either because individuals of the original taxon have not yet been discovered, or because they are extinct. This would not be an exception; the authors of other studies concluded in comparable cases that mitochondrial introgression did cause cyto-nuclear discrepancies, although they failed to identify the source species [13: leaf beetle *Chrysomela*, 56: *Drosophila quinaria*].

Incomplete lineage sorting (ILS) of ancestral polymorphisms is another possible scenario [17, 19] as assumed for example for the mito-nuclear discordance observed in the *Drosophila serido* haplogroup [21]. Distinguishing between ILS and introgression is difficult and often the only indications might be drawn from genetic data. It was suggested that haplotypes which hold a phylogenetically basal position are hints for ancestral polymorphisms and that no predictable geographical pattern is expected from ILS [19]. However, these criteria are inadequate to distinguish ancient mitochondrial introgression from ILS. Most of the previously reported divergent mtDNA lineages in the *D. longispina* complex [8] are phylogenetically basal compared to the mtDNA lineages of well-recognized species and so far no geographic pattern is visible (but the number of observations is very low at the moment). This would support the idea that the mtDNA variation we observed is due to ILS according to the above-mentioned criteria. However, the divergence among mtDNA lineages in this complex [8] is so high (10.8 to 17.6% sequence divergence from their closest sister clade expressed as Kimura 2-parameter distance) that stochastically driven ILS is highly unlikely as lineage sorting in mitochondrial loci progresses fast [19].

Direct balancing selection may preserve ancestral polymorphisms within a population indefinitely [19]. Often, it is assumed that mitochondrial DNA is selectively neutral; however, various recent studies show deviations from these assumptions [e.g. 22, 23, 57] and propose that mitochondrial DNA variation might be determined also by natural selection. Even in the *Daphnia longispina* complex, experimental evidence was collected indicating a higher fitness of certain haplotypes under various temperature regimes [58]. Additionally, simulations have shown that uniparentally inherited loci may display strong phylogeographic structures generated by weak selection [59]. Therefore, it is possible that we detected rare ancestral mitochondrial lineages in the *D. longispina* species complex that are favored under specific environmental conditions, like temperature, predation, food quality or quantity. However, we have no experimental evidence yet to verify the potential adaptive value of the divergent mtDNA lineages.

Besides direct selection, indirect selection induced by maternally inherited symbionts (including parasites), might also explain mito-nuclear discordance [reviewed by 24]. In these cases, certain mitochondrial genomes might be linked to the transmission of those symbionts. The maintenance of different symbionts via natural selection could, therefore, result in mitochondrial differentiation between populations despite ongoing gene flow. However, also selective sweeps, occurring in one population but not in others, might induce this pattern [24]. This is reported for the intracellular bacteria of the genus *Wolbachia* (which is not known to infect *Daphnia* [60]), but a few studies of other vertically transmitted symbionts indicate that this is not genus-specific [24]. Most symbionts so far studied in *Daphnia* (mostly microparasites) are nevertheless transmitted horizontally and not vertically [61], even though many *Daphnia* species, including those of the *D. longispina* complex [e.g., 62], carry them. Intracellular parasites that are known to transmit vertically in *Daphnia* are species of the microsporidian genus *Hamiltosporidium* [63]. Yet, to our knowledge, there is no published study presenting a case of mito-nuclear discordance caused by a microsporidian. Irrespective thereof, the mitochondrial divergence caused by such a process is expected to be lower than the deep divergence we observed in *Daphnia*.

## Conclusions

In the studied populations, we observed high mitochondrial divergence in the absence of nuclear divergence in the *Daphnia longispina* species complex. For the reasons discussed above, we would exclude methodological artifacts (like the amplification and sequencing of numts or pseudogenes instead of the functional gene) as explanation. In addition, the level of divergence among lineages is so high that we find stochastic ILS as well as indirect selection that arose from linkage disequilibrium with maternally transmitted symbionts highly unlikely. Likely explanations are that we observe polymorphisms resulting from past introgression from unknown source taxa, or maintained through selection. Both explanations are plausible, as hybridization and introgression are common phenomena in this species complex [43] and experimental evidence hints that selection of mitochondrial genomes may occur in *Daphnia* [58].

Altogether, we consider past hybridization and introgression as most probable explanation for the observed polymorphisms in the lineages I, II and III, assuming that the original parental lineages did not evolve efficient reproductive barriers. This is commonly observed also for other taxa in the *D. longispina* species complex and long-lasting introgressed lineages have been recorded before; a well-described example is *D. mendotae*, which arose from hybridization between *D. galeata* and *D. dentifera* but subsequently maintained its distinct character [44]. The observed patterns in our study might be the legacy of long-range dispersal events that ended up in mitochondrial DNA introgression rather than establishment of separated lineages. If the presumed parental species of lineages I-III still exist somewhere as entities with distinct nuclear genomes, their distribution is either geographically restricted or scattered [as hypothesized in 8], possibly in understudied regions like Siberia or parts of eastern and north-eastern Europe.

The results of our study illustrate that the discovery of highly divergent mitochondrial DNA lineages does not necessarily translate into the discovery of previously undescribed species. Additional evidence, like the analyses of nuclear markers, morphology, biogeography, or behavior, is mandatory. Detailed studies of divergent mitochondrial lineages frequently reveal relevant, but small, phenotypic differences, supporting the conclusion that they are indeed distinct taxa [e.g., 64, 65]. Also in the genus *Daphnia* two such species have been recently described from Europe: *Daphnia hrbaceki* [66] and *Daphnia inopinata* [67]. Considering the substantial yet undescribed and often unexplored variation within the genus [7], we assume that other new species descriptions will follow. However, our study indicates that high mtDNA divergence represents a precondition, but not a sufficient criterion for species delimitation.

## Additional file

Additional file 1: Mito-nuclear discordance in a *Daphnia* species complex. The additional file contains information on reference populations (**Table S1**), displays the comparison of nuclear and mitochondrial DNA patterns in the *Daphnia longispina* species complex using the first and the third as well as the second and the third factor of a factorial correspondence analysis based on multilocus genotype data from ten microsatellite loci (**Figure S1**), and presents the results of the hierarchical Structure analysis of individuals belonging to *D. longispina*, clade II and III that were assigned to *D. longispina* by the Structure analysis using the whole dataset (**Figures S2** and **S3**). (DOCX 420 kb)
