# Supplementary material for: Divergent clades or cryptic species? Mito-nuclear discordance in a Daphnia species complex
Source: BMC Evol Biol. 2017 Nov 22;17:227. doi: 10.1186/s12862-017-1070-4 (PMC5700674; doi:10.1186/s12862-017-1070-4)
Supplement: Additional file 1: — Mito-nuclear discordance in a Daphnia species complex. The additional file contains information on reference populations (Table S1), displays the comparison of nuclear and mitochondrial DNA patterns in the Daphnia longispina species complex using the first and the third as well as the second and the third factor of a factorial correspondence analysis based on multilocus genotype data from ten microsatellite loci (Figure S1), and presents the results of the hierarchical Structure analysis of individuals belonging to D. longispina, clade II and III that were assigned to D. longispina by the Structure analysis using the whole dataset (Figures S2 and S3). (DOCX 420 kb) [file 12862_2017_1070_MOESM1_ESM.docx]

Additional file 1

**Table S1** **Data of natural reference populations.**

Names of populations are given together with country of origin, corresponding geographical coordinates, population abbreviation, taxon affiliation, and number of individuals used for this study.

| **Population** | **Origin** | **Abbreviation** | **Longitude** | **Latitude** | **Taxon** | **N** |
| --- | --- | --- | --- | --- | --- | --- |
| Mondsee | Austria | AT-MS | 47°50ʹ26ʺ N | 13°22ʹ48ʺ E | *D. longispina* | 17 |
| Beringen | Belgium | BE-BE | 51°00ʹ20ʺ N | 05°17ʹ20ʺ E | *D. galeata* | 14 |
| Diest | Belgium | BE-DI | 50°59ʹ16ʺ N | 05°03ʹ52ʺ E | *D. galeata* | 20 |
| St. Bernard | Switzerland | CH-SB | 45°52ʹ19ʺ N | 07°10ʹ13ʺ E | *D. longispina* | 15 |
| Brno | Czech Republic | CZ-BR | 49°15ʹ36ʺ N | 16°27ʹ18ʺ E | *D. cucullata* | 12 |
| Římov | Czech Republic | CZ-RM | 48°48ʹ25ʺ N | 14°29ʹ30ʺ E | *D. galeata* | 13 |
| Stanovice | Czech Republic | CZ-ST | 50°10ʹ30ʺ N | 12°53ʹ00ʺ E | *D. galeata* | 18 |
| Vranov | Czech Republic | CZ-VR | 48°54ʹ28ʺ N | 15°49ʹ01ʺ E | *D. longispina* | 12 |
| Helgoland | Germany | DE-HL | 54°11ʹ04ʺ N | 07°54ʹ46ʺ E | *D. longispina* | 17 |
| Ismaning | Germany | DE-IS | 48°13ʹ15ʺ N | 11°46ʹ19ʺ E | *D. longispina* | 14 |
| Stechlinsee | Germany | DE-SS | 53°09ʹ00ʺ N | 13°01ʹ58ʺ E | *D. longispina* | 19 |
| Usingen | Germany | DE-US | 50°20ʹ37ʺ N | 08°30ʹ26ʺ E | *D. cucullata* | 11 |
| Cogollos | Spain | ES-CO | 37°12ʹ36ʺ N | 02°50ʹ06ʺ W | *D. galeata* | 17 |
| Lake Pyhäjärvi | Finland | FI-PY | 62°17ʹ31ʺ N | 26°46ʹ08ʺ E | *D. galeata* | 14 |
| Lake Vesijärvi | Finland | FI-VJ | 60°59ʹ38ʺ N | 25°37ʹ45ʺ E | *D. cucullata* | 13 |
| Loch Leven | Great Britain | GB-LO | 56°12ʹ00ʺ N | 03°22ʹ48ʺ W | *D. galeata* | 13 |
| Rollesby | Great Britain | GB-RO | 52°41ʹ20ʺ N | 01°39ʹ24ʺ E | *D. galeata* | 12 |
| Mývatn | Iceland | IS-MY | 65°34ʹ56ʺ N | 16°59ʹ22ʺ W | *D. galeata* | 13 |
| Lake Asveja | Lithuania | LT-AS | 55°02ʹ42ʺ N | 25°30ʹ06ʺ E | *D. longispina* | 9 |
| Luodis | Lithuania | LT-LU | 55°34ʹ54ʺ N | 26°12ʹ44ʺ E | *D. cucullata* | 10 |
| Lake Smartinsko | Slovenia | SI-SJ | 46°16ʹ58ʺ N | 15°15ʹ57ʺ E | *D. cucullata* | 14 |
| Reservoir Dubník II | Slovakia | SK-DU | 48°46ʹ01ʺ N | 17°40ʹ58ʺ E | *D. cucullata* | 15 |
| Total |  |  |  |  |  | 312 |


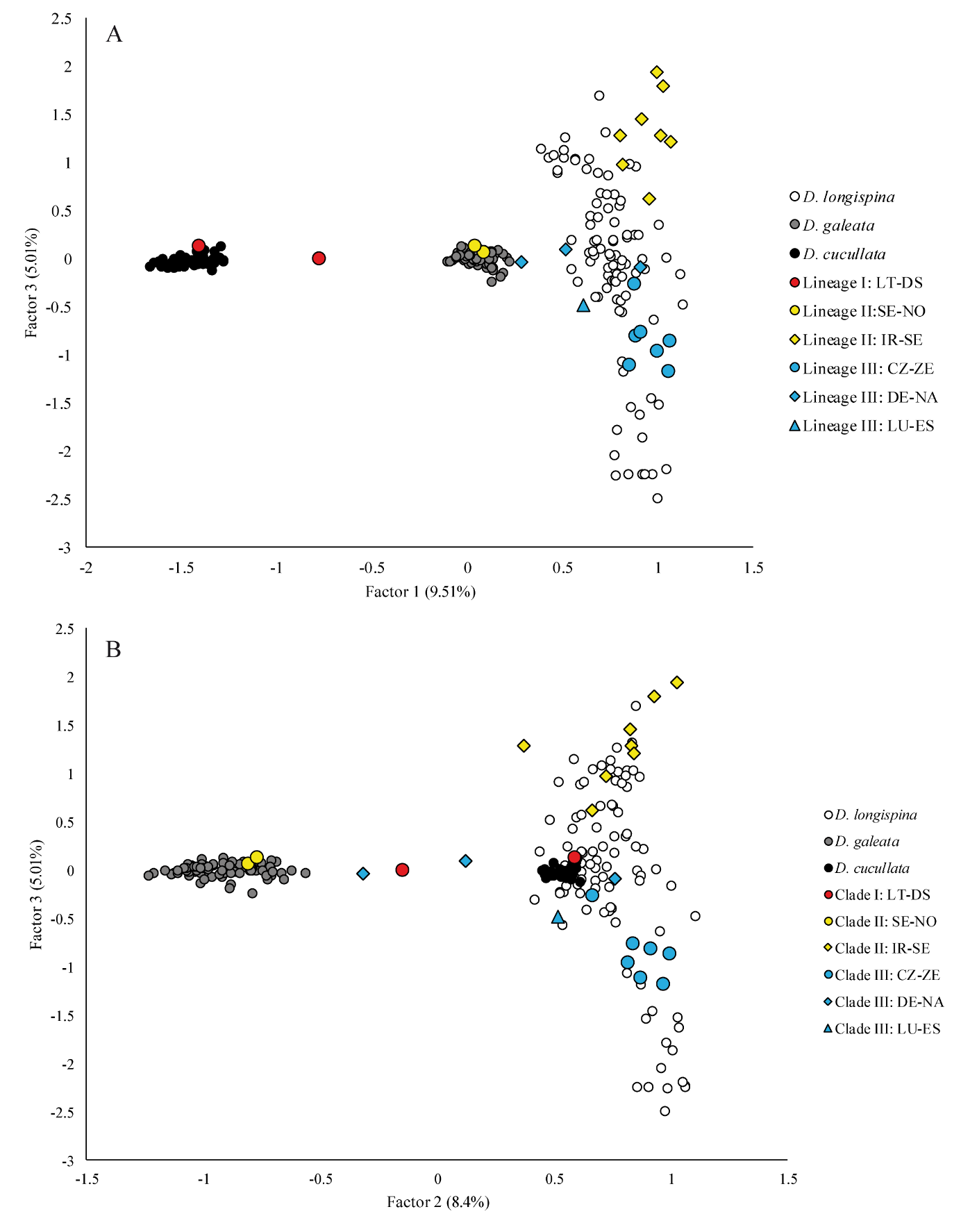


**Figure S1 Comparison of nuclear and mitochondrial DNA patterns in the *Daphnia longispina* species complex.**

Factorial correspondence analysis demonstrating the position of the 49 individuals belonging to mtDNA clades I, II, or III in relation to the reference dataset consisting of 312 individuals belonging to *D. galeata* (grey circles), *D. longispina* (white circles), and *D. cucullata* (black circles). Genetic relationships are depicted using (A) the first and the third factor, or (B) the second and the third factor of an FCA based on multilocus genotypes from ten microsatellite loci.

**Figure S2 Detection of the uppermost hierarchical level of genetic structure in the microsatellite dataset consisting of all individuals assigned to *D. longispina*.**

Mean LnP(*K*) and convergence between replicates (open circles with error bars) as well as Delta *K* (filled circles) were used for the detection of the most likely number of *K*. The graph is modified from the output derived from Structure Harvester [36]. According to these results, *K* = 7 or *K* = 8 is adequate to describe the structure in the dataset.

**
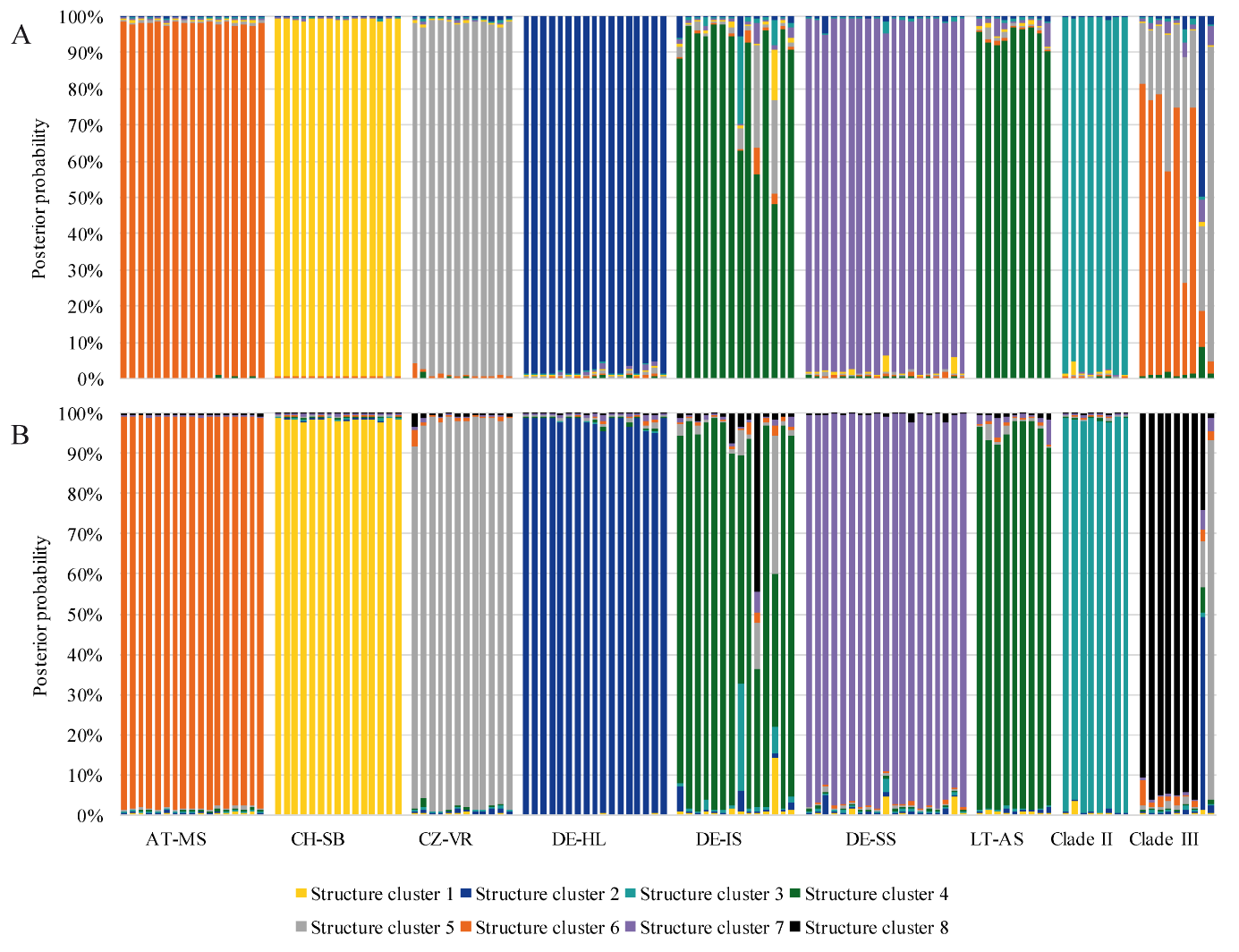
**

**Figure S3 Results of the admixture analysis of all *D. longispina* reference individuals (N = 103) and the individuals belonging to mtDNA clades II (N = 8) and III (N = 9) that were assigned to *D. longispina* by the Structure analysis using the whole dataset (see main text).**

Shown are the results of (A) *K* = 7 and (B) *K* = 8 where almost each cluster associates to one of the geographical populations (populations DE-IS and LT-AS are grouped in one Structure cluster). Clade III contains individuals from 3 localities which are grouped to different Structure clusters. The population identification codes are given on the x-axis and the posterior probabilities on the y-axis.
